# Exploring the binding efficacy of ivermectin against the key proteins of SARS-CoV-2 pathogenesis: an *in silico* approach

**DOI:** 10.2217/fvl-2020-0342

**Published:** 2021-03-25

**Authors:** Abhigyan Choudhury, Nabarun C Das, Ritwik Patra, Manojit Bhattacharya, Pratik Ghosh, Bidhan C Patra, Suprabhat Mukherjee

**Affiliations:** 1 ^1^Integrative Biochemistry & Immunology Laboratory, Department of Animal Science, Kazi Nazrul University, Asansol 713340, West Bengal, India; 2 ^2^Department of Zoology, Fakir Mohan University, Balasore 756020, Odisha, India; 3 ^3^Department of Zoology, Vidyasagar University, Midnapore 721102, West Bengal, India

**Keywords:** ivermectin, molecular docking, protease, replicase, SARS-CoV-2, spike glycoprotein

## Abstract

**Aim:** COVID-19 is currently the biggest threat to mankind. Recently, ivermectin (a US FDA-approved antiparasitic drug) has been explored as an anti-SARS-CoV-2 agent. Herein, we have studied the possible mechanism of action of ivermectin using *in silico* approaches. **Materials & methods:** Interaction of ivermectin against the key proteins involved in SARS-CoV-2 pathogenesis were investigated through molecular docking and molecular dynamic simulation. **Results:** Ivermectin was found as a blocker of viral replicase, protease and human TMPRSS2, which could be the biophysical basis behind its antiviral efficiency. The antiviral action and ADMET profile of ivermectin was on par with the currently used anticorona drugs such as hydroxychloroquine and remdesivir. **Conclusion:** Our study enlightens the candidature of ivermectin as an effective drug for treating COVID-19.

The Coronaviridae family of viruses has engraved its name in history by cursing humankind with three major blows – the severe acute respiratory syndrome (SARS) caused by SARS-CoV, middle east respiratory syndrome (MERS) caused by MERS-CoV, and the latest pandemic outbreak in the form of COVID-19 caused by SARS-CoV-2 [1]. Hitherto, there are over 21.29 million confirmed cases of COVID-19 globally, which has already taken 0.76 million lives till the mid of August 2020 [2]. The SARS-CoV-2 belongs to the β-Coronavirus genus of a 2B group of the Coronaviridae family. This viral strain consists of four major structural proteins such as S protein, which encompasses the spike, E forming the envelope, M for the membrane and N for the nucleocapsid. This nucleocapsid contains a 29,903 base long, positive-sense single-stranded RNA genome [3]. The virus transmits from person to person mainly via close physical contact and by respiratory aerosols which are produced during coughing, sneezing, and even talking [4]. It is well assumed that the virus may also spread via fecal matters and by fomite transmission, which occurs when a person comes into contact with a contaminated surface [5]. Unrestricted domestic and international air travels from COVID hotspots are also considered significant contributors to the global spread of this viral infection [6].

To date, several postulations are available regarding the mechanism of the pathogenesis of the virus in a human host. However, the actual mechanistic pathway is still undefined. In general, aerosol droplets containing the virus particle gains access into the human respiratory system, precisely the alveolar membranes [7]. After its entry into the respiratory system, the spike glycoprotein ectodomain present on the viral capsid binds to the angiotensin-converting enzyme-2 (ACE2) transmembrane receptor protein, consequently, the RNA genome enters the alveolar cells by receptor-mediated endocytosis [8]. The viral RNA-dependent RNA polymerase (RDRP; replicase) is eventually translated from its mRNA strand with the help of its main protease enzyme, and the replicase enzyme catalyzes the rapid replication of the viral genome alongside other structural proteins required for reconstructing new viral particles [8]. In addition to these, interactions between the viral antigens and host immune cells are considered as a crucial determinant factor of the immunopathological attributes of COVID-19 [9]. Proinflammatory responses induced from host–virus interactions trigger vasodilation, accumulation of humoral factors that ultimately result in fever, abnormal alveolar exchange and breathing difficulty, leading to death of patients [10].

While the pandemic is spreading faster than wildfires, the unavailability of ratified drugs and or vaccine against the same has made the situation more alarming. In this context, recent studies on the use of hydroxychloroquine (an antimalarial drug) in combination with the antibiotic azithromycin [11] and antiretroviral drugs like remdesivir, EIDD-2801 or favipiravir have shown effectiveness against SARS-CoV-2 [12]. Based on this, ivermectin has been recently reported as the most active agent against COVID-19 among the US FDA-approved drugs *in vitro* trial [13]. Ivermectin is a macrocyclic lactone natively used to treat a broad spectrum of parasitic infestations including lymphatic filariasis and onchocerciasis [14]. Interestingly, a recent study claims that the drug inhibits the replication of SARS-CoV-2 in *in vitro* condition and can reduce the spread of the virus by approximately 5000-times within 48 h while being tested *in vitro* using primate cell lines [13]. Considering the therapeutic promise of ivermectin against COVID-19 [15], the present study has been conducted to represent the efficacy of this drug against the four most crucial functional proteins of SARS-CoV-2 using advanced biocomputational approaches. Moreover, the efficacy of ivermectin has been compared with two of the recently used anticorona drugs, namely hydroxychloroquine and remdesivir.

## Materials & methods

### Data mining

The commercial ivermectin formulation is comprised of a racemic mixture of -O-dimethyl-22,23-dihydroavermectin B1a (ivermectin B1a) and 5-O-dimethyl-22,23-dihydroavermectin B1b (ivermectin B1b) and both structures were used in this study. 3D structures of ivermectin homologs, hydroxychloroquine and remdesivir were retrieved from PubChem compound library (https://pubchem.ncbi.nlm.nih.gov/). The structures were converted in .pdb format for further use. The structure of each ligand of ivermectin obtained from the Pubchem library was converted to 3D conformer (Supplementary Figure 1A) with minimal energy using Frog2 server. The 3D conformers of both remdesivir and hydroxychloroquine were downloaded from PubChem library. All these 3D conformers were used in protein–ligand docking study.

Full-length amino acid sequences of human ACE2 receptor protein (Accession ID: AAT45083.1), Human TMPRSS2 (Accession ID: AAH51839.1), SARS-CoV-2 Spike S1 receptor-binding domain (RBD; Accession ID: pdb|6M17|F) and SARS-CoV-2 NSP9 replicase enzyme (Accession ID: pdb|6W4B|A) were retrieved from NCBI protein database (www.ncbi.nlm.nih.gov). Furthermore, the crystal structure of SARS-CoV-2 protease (Protein Data Bank [PDB] ID: 6Y2E [DOI: 10.2210/pdb6Y2E/pdb]) was obtained from the RCSB PDB (www.rcsb.org). The crystal structure was generated *ab initio* by using x-ray diffraction techniques with a resolution of 1.75Å. A resolution below 3.0Å suggests good structural detailing which is desirable for molecular docking studies. This structure was introduced to PyMOL software application, whereby water molecules present in the original crystal structure were separated and removed from the native structure of the protein such to avoid undesirable interferences. On the other side, the structure of S2 subunit of spike protein was separately modeled by using the amino acid sequence of S2 and PDB ID 6VYB as a template. Crystal structure of the SARS-CoV-2 in native form, the RDRP was acquired from PDB (ID: 6M71). 3D structure of target proteins from SARS-CoV-2 and humans are represented in Supplementary Figure 1B–H.

### Homology modeling & model validation

3D structures of the target proteins/peptides were built through homology modeling strategy using the MODELLER software (Ver. 9.24 x64 Windows). The stereochemical qualities of the generated models were assessed by determining Ramachandran Plots using the structural assessment tool provided by the SWISS-MODEL web server (https://swissmodel.expasy.org/).

### Molecular docking & visualization

The solved molecular structures obtained from PDB afterword homology modeling were subjected to protein–ligand docking using Hex 8.0.0 software package. Hex 8.0.0 is a Fourier transform (FFT)-based protein docking program wherein receptor and ligand structures were fed into the program in terms of PDB files for interaction based on the shape and electrostatic correlation parameters. The output of the docking study for each experiment was also subjected to postprocess analysis using optimized potentials for liquid simulations force field minimization for optimizing the global E_Tot_ output. The energy values (E-values) were recorded for each output docked complex. The structures of the docked receptor–ligand complexes were later rendered and visualized using the Visual Molecular Dynamics software suite (Ver. 1.9.3) and further interpreted accordingly.

### Drug–target interactions

Protein–drug interactions were determined by analyzing the docked complexes using Protein Ligand Interaction Profiler (PLIP) server (https://projects.biotec.tu-dresden.de/). PLIP is a Python-based open source software that provides a complete analysis and visualization of the noncovalent protein–ligand interactions even on single-atom level that includes seven prime interaction types like hydrogen bonds, hydrophobic contacts, π-stacking, π–cation interactions, salt bridges, water bridges and halogen bonds.

### Molecular dynamic simulation

Molecular dynamics of strongly docked complexes among the drugs and target proteins (e.g., ivermectin and SARS-CoV-2 protease or ivermectin and human ACE2 [hACE2] receptor) were performed through iMODS server to explain the usual protein motion within the internal coordinates through normal mode analysis (NMA) [16]. iMODS is a user-friendly, highly customizable server and discloses several coarse-grained (CG) levels. The server calculates the dihedral coordinates of Cα atoms of large macromolecules. Moreover, the iMODS calculates B-factor, structural deformability and calculates the eigenvalue.

### Determination of binding free energy

The highest active molecule of ivermectin B1b was examined for its binding efficacy against the most favorable target – the RDRP. Binding free energy was calculated using AutoDock software using the following formula:

$$\text{Conversion}\;\text{formula}=Kd={\text{e}}^{\left\{\frac{\Delta G\times 1000}{RT}\right\}}$$

### *In silico* analysis of pharmacokinetics

Comparative pharmacokinetic attributes like absorption, distribution, metabolism and excretion (ADME) and cytotoxicity as well as other important pharmacological properties (physicochemical properties, lipophilicity, water-solubility and drug-likeness) of the three drugs of choice were analyzed employing SwissADME open-access server (www.swissadme.ch/).

## Results

### Molecular docking studies

In the present study, molecular docking was used to explore the targets of ivermectin in SARS-CoV-2 and to determine the comparative therapeutic efficacy with hydroxychloroquine and remdesivir, which are currently in use for treating COVID-19. While working with the molecular models, the quality of emulation of the molecular mechanics is known to depend on the feature of the models used for docking [17]. Therefore, we checked the stereochemical quality of each model. It was found that all the models had more than 92% of residues in favored regions, and it may indicate an optimal stereochemical quality that can be used for further studies (Supplementary Figure 2). Docking studies conducted using Hex provides E-value for every binding conformation, which is just inversely proportionate to the binding efficiency of the structure characterized by negative E-value. Suspiring confidence from the above assessment, protein–ligand docking studies were performed to gain insight into the most probable and efficient binding conformations of ivermectin with the proteins of interest. The results have been furnished in the subsequent subsections mentioned in the below.

### Interaction of ivermectin with the spike glycoprotein of SARS-CoV-2

**Figure 1.**
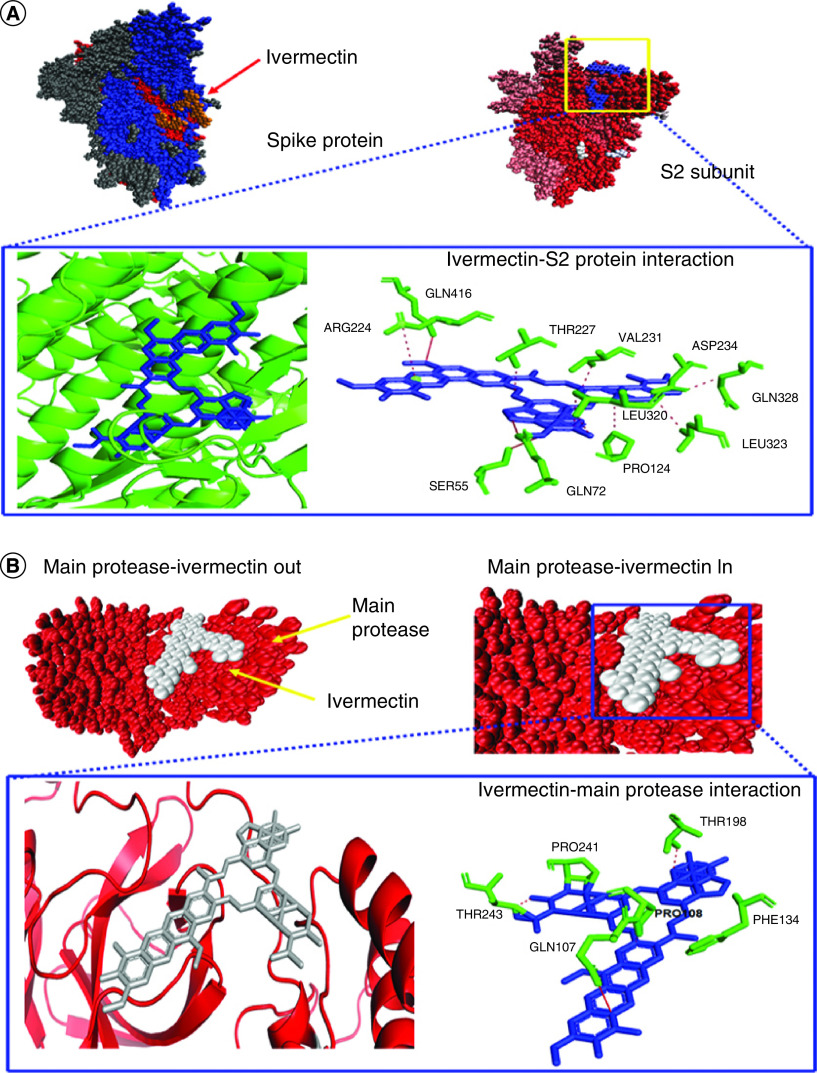
Molecular interactions among S2 subunit of spike protein and main protease with ivermectin, respectively. Molecular docking poses exhibiting binding conformation in space-filling models and noncovalent interactions analyzed by PLIP server, among **(A)** S2 subunit protein and ivermectin and **(B)** main protease and ivermectin. PLIP: Protein–ligand interaction profiler.

Our experimental data on the docking of ivermectin on SARS-CoV-2 spike protein (in native form) revealed a strong binding of the compound with an energy value of -261.74 and -287, respectively, for B1a and B1b homologs. Spike protein is a homotrimeric protein with two functional S1 subunits and one structural S2 subunit [18]. Therefore, we checked the actual binding site of ivermectin isomers in the spike protein through separate docking using S1 and S2 subunits. Results of molecular docking using the Hex software program are shown in Figure 1A and Table 1. It was observed that the ivermectin homologs can bind with both S1 (the receptor-binding domain of the spike protein) and S2 subunits of the SARS-CoV-2 spike protein. But, the strength of the binding of ivermectin isomers were more intense on the S2 subunit (Figure 1A & Table 1). Energy value (E_Tot_- values) for the interaction of B1a and B1b were -372.99 and -393.29 for S1 protein while -395.9 and -411.6. Therefore, it may be inferred that binding of ivermectin at S2 subunit of spike protein may cause an allosteric effect, which in turn can induce a conformational change in the whole protein or receptor-binding S1 subunit. Ivermectin B1a has been found to be the better molecule in targeting spike protein or its subunits than B1b isomer. We also scrutinized the stability of ivermectin-SARS-CoV-2 spike protein complex through molecular docking analysis stated in the later part of the manuscript.

**Table 1.** Protein–ligand interactions among ivermectin homologs and SARS-CoV-2 spike protein.

S1 receptor-binding domain							
Ivermectin B1a				Ivermectin B1b			
Hydrophobic interaction							
Residue	Distance (Å)	Ligand atom no.	Protein atom no.	Residue	Distance (Å)	Ligand atom no.	Protein atom no.
TYR51	3.87	6618	513	VAL185	3.01	6625	6174
ALA54	3.57	6593	542	TYR187	2.94	6624	4009
LYS60	3.27	6620	599				
PRO66	3.61	6618	653				
GLU88	3.58	6644	5224				
ARG90	3.51	6643	5241				
TYR187	3.71	6633	6190				

Hydrogen bonding							
Residue	Distance (Å)	Ligand atom no.	Protein atom no.	Residue	Distance (Å)	Ligand atom no.	Protein atom no.
SER55	2.62	6639	547	SER57	3.26	6618	4927
ASP87	4.09	6584	5217	ASN119	4.06	6621	5521
THR97	2.80	6616	5310				
TYR187	3.18	6641	6192				

Salt bridges							
Residue	Distance (Å)	Ligand atom no.	Protein atom no.				
ARG85	3.37	6585, 6584	5156				
ARG90	4.00	6605, 6607	5241				

S2 subunit							
Ivermectin B1a				Ivermectin B1b			
Hydrophobic interaction							
Residue	Distance (Å)	Ligand atom no.	Protein atom no.	Residue	Distance (Å)	Ligand atom no.	Protein atom no.
THR182	3.00	16,986	7255	ARG224	3.25	16,964	13,252
GLU239	3.88	17,001	13,395	ALA225	3.59	16,957	13,267
LYS245	3.86	17,004	13,453	VAL231	3.96	16,960	13,312
ALA485	3.13	17,001	15,696	GLU232	3.90	16,963	13,319
ASP500	3.94	16,948	10,223	ASP409	2.99	16,980	9358
				GLN413	3.42	16,967	9395

Hydrogen bonding							
Residue	Distance (Å)	Ligand atom no.	Protein atom no.	Residue	Distance (Å)	Ligand atom no.	Protein atom no.
THR183	4.01	16,983	7258	ARG224	4.06	16,992	13,253
GLU239	3.80	17,000	13,399	GLU232	4.06	16,998	13,315
ASP500	3.83	16,945	10,226	ASN412	3.92	17,001	9386
LYS504	4.08	16,960	10,252	GLN413	3.47	16,996	9398
				GLU476	3.81	16,997	10,002

SARS-CoV-2 main protease							
Ivermectin B1a				Ivermectin B1b			
Hydrophobic interaction							
Residue	Distance (Å)	Ligand atom no.	Protein atom no.	Residue	Distance (Å)	Ligand atom no.	Protein atom no.
THR25	3.35	3258	221	THR169	3.70	2924	1605
MET165	3.80	3211	1565	ASN238	2.67	2946	2263
GLU166	3.56	3243	1579	TYR239	3.88	2933	2278
GLU166	3.43	3219	1578	LEU287	3.70	2944	2700
GLN189	3.78	3209	1787				

Hydrogen bonding							
Residue	Distance (Å)	Ligand atom no.	Protein atom no.	Residue	Distance (Å)	Ligand atom no.	Protein atom no.
ASN142	3.12	3259	1357	THR169	3.79	2923	1606
GLY143	3.94	3202	1361	ALA194	3.77	2923	1827
GLU166	3.43	3217	1574	GLY195	2.61	2920	1833
				TYR239	2.72	2945	2281
				LEU287	3.67	2694	2889

Replicase							
Ivermectin B1a				Ivermectin B1b			
Hydrophobic interaction							
Residue	Distance (Å)	Ligand atom no.	Protein atom no.	Residue	Distance (Å)	Ligand atom no.	Protein atom no.
PRO87	3.92	2173	732	ASP51	2.88	2180	384
LYS88	3.12	2144	741	LYS88	3.03	2176	740
				LYS88	3.12	2150	741
				VAL89	3.74	2178	753

Hydrogen bonding							
				ASP51	4.04	2189	380
				VAL89	3.87	2157	748

RNA-dependent RNA polymerase							
Ivermectin B1A				Ivermectin B1B			
Hydrophobic interaction							
Residue	Distance (Å)	Ligand atom no.	Protein atom no.	Residue	Distance (Å)	Ligand atom no.	Protein atom no.
ASN496	3.69	10,471	4272	ASN496	3.81	10,450	4272
LYS500	3.99	10,461	4314	LYS500	3.04	10,431	4313
VAL557	3.76	10,457	4900	LYS545	3.30	10,460	4771
ALA685	3.91	10,464	6107	VAL557	2.96	10,459	4900

Hydrogen bonding							
Residue	Distance (Å)	Ligand atom no.	Protein atom no.	Residue	Distance (Å)	Ligand atom no.	Protein atom no.
ASN496	2.60	10,470	4275	ASN497	3.68	10,452	4286
ASN497	3.33	10,468	4286	LEU544	3.58	10,471	4760
LYS500	2.83	10,472	4316	TYR546	3.60	10,471	4779
ILE548	3.54	10,450	10,450				
ARG555	4.06	10,450	4878				

Salt bridges							
Residue	Distance (Å)	Ligand atom no.	Protein atom no.				
LYS500	5.37	10,415; 10,416	4314				
LYS545	4.65	10,436; 10,438	4762				
LYS545	3.97	10,441; 10,443	4762				
ARG555	5.03	10,441; 10,443	4878				

Human TMPRSS2 protein							
Ivermectin B1a				Ivermectin B1b			
Hydrophobic interaction							
Residue	Distance (Å)	Ligand atom no.	Protein atom no.	Residue	Distance (Å)	Ligand atom no.	Protein atom no.
PRO369	3.78	3336	2167	VAL298	3.67	3337	1468
ASP482	3.16	3368	3210	VAL331	3.99	3334	1799
TRP483	3.79	3361	3219	VAL331	2.64	3337	1800
				SER333	2.49	3340	1816

Hydrogen bonding							
Residue	Distance (Å)	Ligand atom no.	Protein atom no.	Residue	Distance (Å)	Ligand atom no.	Protein atom no.
ASP220	4.05	3342	751	VAL331	3.62	3381	1798
ASP482	3.90	3365	3213				
ASP482	3.61	3365	3206				
TRP483	4.02	3353	3215				

Salt bridges							
Residue	Distance (Å)	Ligand atom no.	Protein atom no.	Residue	Distance (Å)	Ligand atom no.	Protein atom no.
ARG486	5.47	3353, 3355	3251				

### Interaction of ivermectin with SARS-CoV-2 main protease

After checking the interaction with spike protein, we examined the efficacy of ivermectin against the main viral protease. The results of molecular docking following protein–ligand interaction are given in Figure 1B and Table 1. We have documented an intense binding of both ivermectin B1a and B1b isomer to the main protease with subsequent energy (E_Tot_-) values of -384.56 and -408.6. To explore the interacting residues from the target, a docked complex comprising protease and ivermectin was analyzed by PLIP tool. It was observed that Pro108, Phe134, Thr198, Pro241 and Thr243 residues from SARS-CoV-2 protease were involved in forming hydrophobic interactions with ivermectin, while the Gln107 was involved in hydrogen bonding with the ligand element – the B_1B_ homolog (Figure 1B & Table 1).

### Interaction of ivermectin with SARS-CoV-2 replicase & RDRP

**Figure 2.**
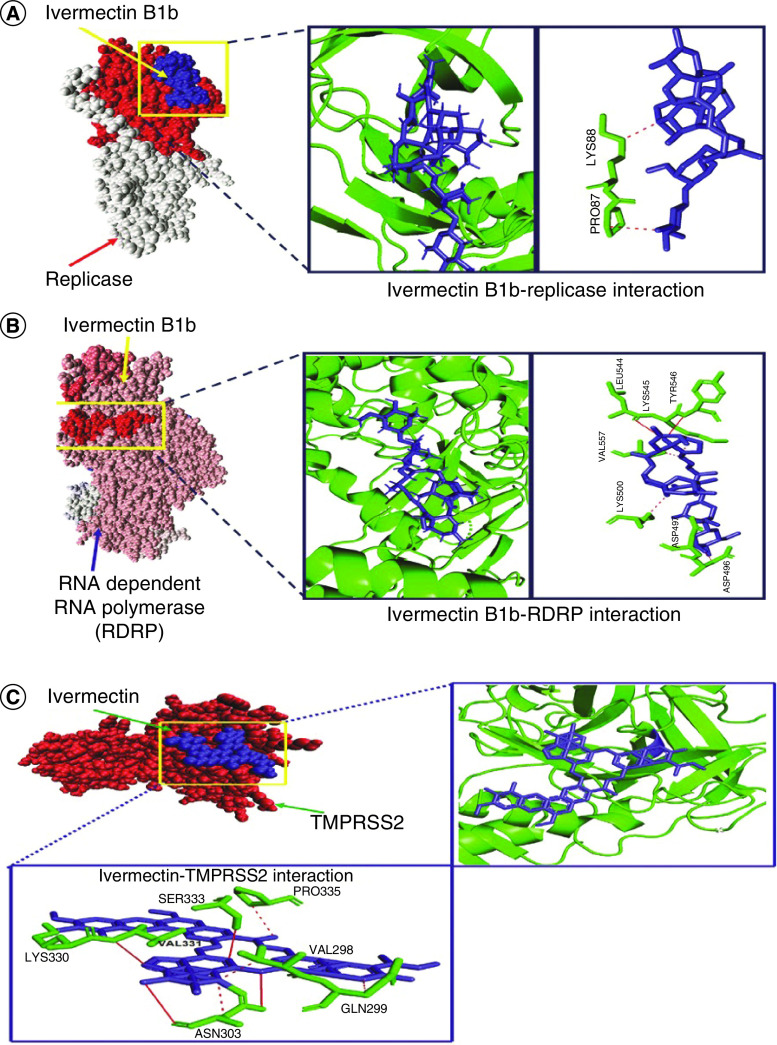
Binding interactions of viral replicase, RNA-dependent RNA polymerase and human TMPRSS2 with ivermectin. Docking configurations showing binding modes in space-filling models and noncovalent interactions analyzed by PLIP server between **(A)** viral replicase protein and ivermectin B1b homolog, **(B)** RDRP and ivermectin B1b homolog and **(C)** ivermectin and human TMPRSS2. PLIP: Protein–ligand interaction profiler; RDRP: RNA-dependent RNA polymerase.

Ability of transcribing RNA using replicase and/or RDRP is one of the unique pathogenic hallmarks of SARS-CoV-2. In this connection, we have investigated whether the ivermectin could bind to RNA-synthesizing machinery, in other words, the viral replicase and/or RDRP enzyme or not. Our data revealed that the 5-O-dimethyl-22,23-dihydroavermectin B1a and ivermectin B1b homologs are able to bind with viral replicase (NSP9) with respective energy value of -327.47 and -352.2 (Table 1). Furthermore, we have also found that this strong interaction between replicase and ivermectin is due to intense binding of ivermectin at the RDRP domain (Figure 2B). Ivermectin B1b isomer was found to be the better molecule to form strong interaction with both replicase and which revealed very weak interaction with ivermectin though both of the ivermectin isomers were found to interact with the target protein (Figure 2A–B & Table 1). Major interacting residues of ivermectin forming noncovalent bonds with replicase and RDRP are presented in Figure 2A–B. Alike other protein targets, the binding affinity of ivermectin B1b to replicase and/or RDRP was higher than the binding of ivermectin B1a (Figure 2A & B).

### Interaction of ivermectin with human ACE2 receptor protein

SARS-CoV-2 spike protein objects to utilize human ACE2 for binding and viral entry. However, ACE2 is also important for maintaining the normal physiological function in the human body. Molecular docking of ivermectin with the ACE2 protein displayed weak binding of ivermectin B1a (E-value: -81.85) and B1b (E-value: -91.4) (Table 1; Supplementary Table 1 & Supplementary Figure 3). Studies on the protein–ligand interactions of the docking complexes further explored hACE2-Ivermectin is principally regulated by hydrophobic interactions wherein Asp299, Val298 and Ala301 residues of hACE2 were primarily involved (Supplementary Table 1). The inference obtained from molecular docking was further verified by simulation dynamics (stated in the subsequent section).

### Interaction of ivermectin with human TMPRSS2 receptor protein

TMPRSS2 performs a crucial role in the ACE2-mediated entry in human cells and pathogenesis of SARS-CoV-2. Therefore, TMPRSS2 could be a therapeutic target and we have studied the interaction between ivermectin and TMPRSS2 protein. As depicted in Table 1, ivermectin B1a and B1b were found to bind with TMPRSS2 with respective energy value of -392.75 and -382.9. Binding of ivermectin is majorly orchestrated by the formation of hydrogen bonds and hydrophobic interactions (Figure 2C & Table 1). Interestingly, the binding of ivermectin to hTMPRSS2 also revealed that ivermectin preferably targets binding zone when S1 protein occupies [19]. Such a strong interaction indicated toward the potential of ivermectin to disrupt host–virus interaction. Stability of the interaction was verified by molecular dynamic simulation.

### Molecular dynamic simulation

**Figure 3.**
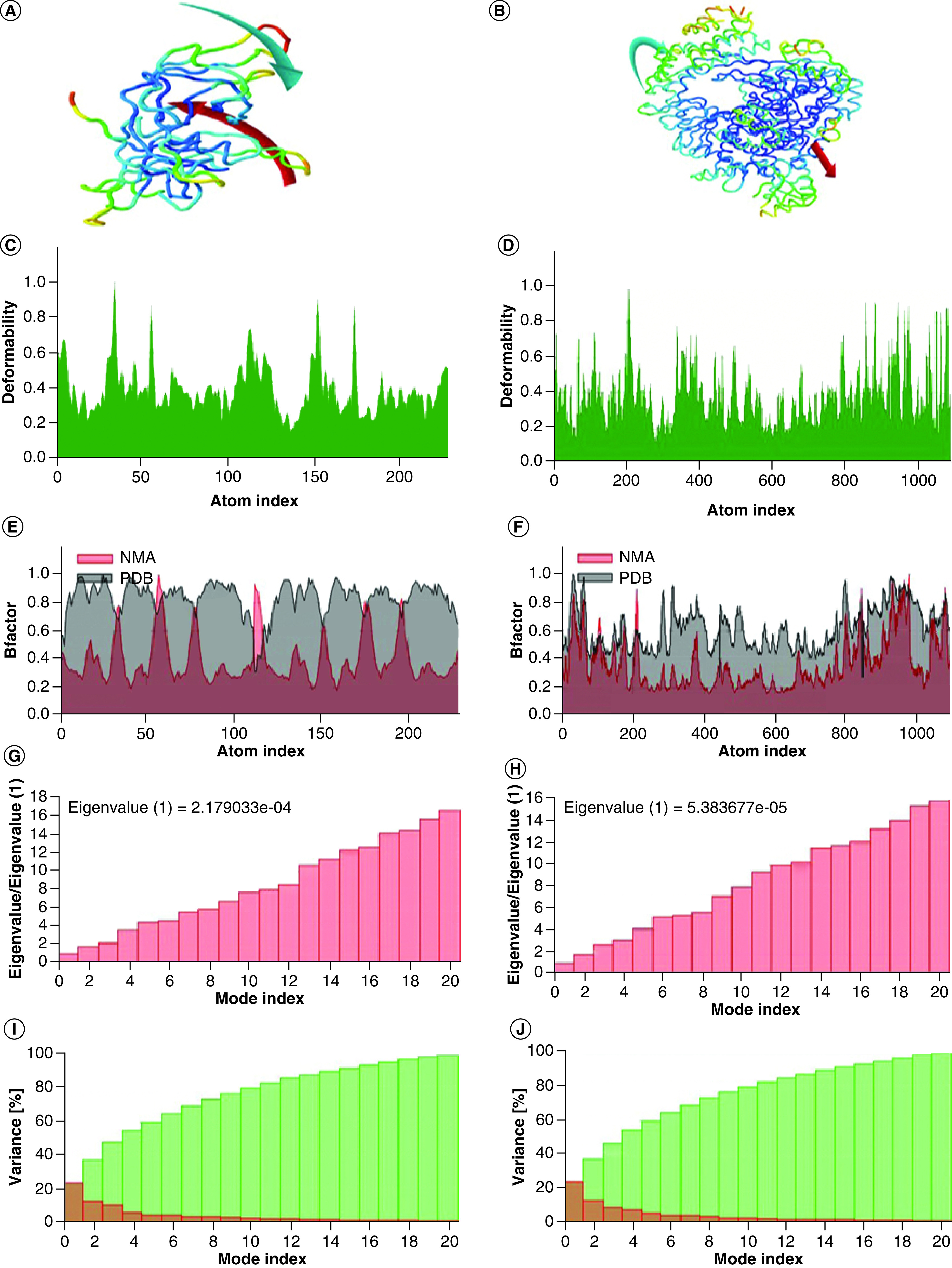
Molecular dynamics simulation analyses of the ivermectin-replicase and ivermectin-RNA-dependent RNA polymerase interactions. Results of molecular dynamics simulation of ivermectin and SARS-CoV-2 replicase and ivermectin and RDRP protein, respectively **(A & B)** NMA mobility, **(C & D)** deformability, **(E & F)** B-factor, **(G & H)** eigenvalues, **(I & J)** variance (red color remarks individual variances and green color indicates cumulative variances). RDRP: RNA-dependent RNA polymerase.

Study of molecular dynamics played a critical role to validate the protein–ligand binding, which can be demonstrated by comparing protein dynamics of their normal mode. In the current study, essential molecular dynamics was also employed to the selected number of normal modes of protein to determine its stability and mobility through iMODS server. We have studied the dynamics of binding of two significant docked complexes comprising ivermectin B1b-viral replicase and ivermectin B1b-RDRP (Figure 3). The result of iMODs directed the domains mobility toward each other presented as arrows in Figure 3A and B showed the 3D interaction models of ivermectin B1a-SARS-CoV-2 replicase and ivermectin B1b-replicase complexes. The B-factor values revealed the relative amplitude of atomic displacements around the equilibrium state and also inferred via NMA was equivalent to RMS (Figure 3E & F). While deformability calculation based on the individual distortion of each residue, hinges of the plot represent high deformability region of the chain (Figure 3C & D). The motion stiffness of Cα atoms calculated through eigenvalue along with verified normal mode model, lower eigenvalue indicated the easier the deformation as lower energy is required to deform the complex structure. The respective eigenvalues for ivermectin B1a-SARS-CoV-2 replicase complex and ivermectin B1a-SARS-CoV-2 replicase complex were found to be 2.179033 × 10^-4^ and 7.466426 × 10^-7^, respectively, that indicated very high stability of the complexes (Figure 3G & H). The variance allied to each normal mode (here 20 normal modes were selected for calculation) that is inversely related to the eigenvalue, individual variances represented by red colors and cumulative variances indicated by green color are shown in Figure 3I and J. The covariance matrix showed the connection between pairs of residues, as the red, white and blue colors indicate the correlated, uncorrelated and anticorrelated pairs of residues, respectively, shown in Supplementary Figure 4A & B. Whereas an elastic network graph represents the pairs of atoms connected by springs and each dot in the graph characterized one spring between the corresponding pair of atoms. In the graph, darker grays specify stiffer springs shown in Supplementary Figure 4C & D.

### Comparing the efficacy of ivermectin with hydroxychloroquine & remdesivir

**Figure 4.**
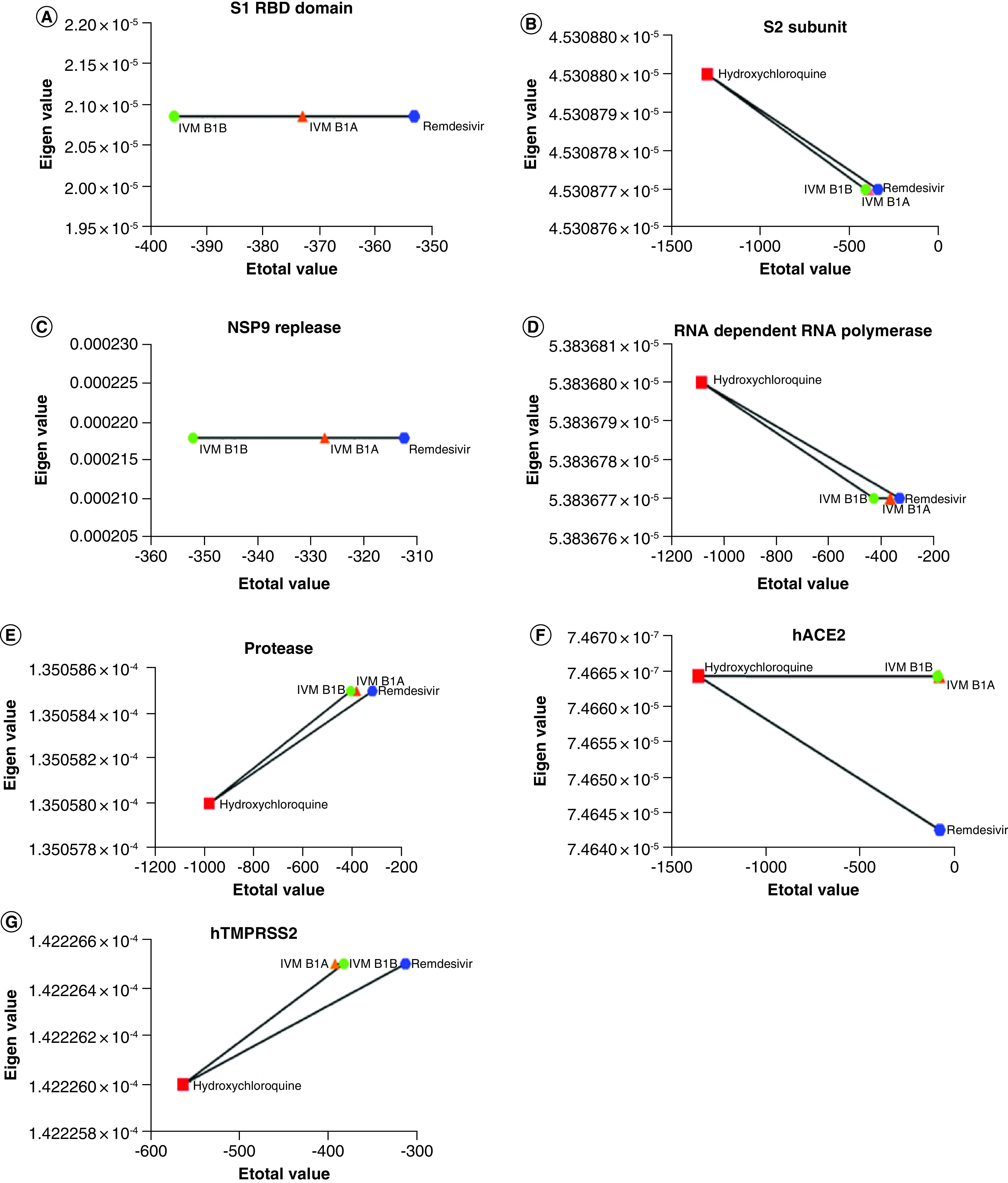
Comparative molecular dynamics simulation analyses of the interactions of ivermectin, hydroxychloroquine and remdesivir with the viral proteins, human ACE2 and TMPRSS2. **(A)** Binding dynamics of ivermectin isomers and remdesivir to viral S1 RBD. **(B)** Comparative binding dynamics of ivermectin isomers, remdesivir and hydroxychloroquine to viral S2 protein. **(C)** Dynamic changes in the binding of ivermectin isomers and remdesivir to NSP9 replicase of SARS-CoV-2. Eigen values showing binding stability of ivermectin isomers, remdesivir and hydroxychloroquine to **(D)** RNA-dependent RNA polymerase and **(E)** protease enzymes of the virus. Comparative binding efficacy of ivermectin isomers, remdesivir and hydroxychloroquine to **(F)** ACE2 receptor and **(G)** TMPRSS2 receptor of human.

Since the synthetic pharmacological formulation of ivermectin essentially consists of a mixture of two homologs – Ivermectin B_1a_ (≥80%) and B_1a_ (≤20%), we checked the effects of the homologs in the mixture and separately (Table 2). Our data indicated that homolog B1b is more effective than B1a. We have further compared the *in silico* efficacy of ivermectin with hydroxychloroquine and remdesivir in terms of binding to the key proteins involved in the pathogenesis of SARS-CoV-2 (Table 2). Our molecular docking data suggest that ivermectin possesses a better potential than remdesivir to bind with spike protein, RBD, S2 subunit and RDRP of SARS-CoV-2 (Table 2). However, hydroxychloroquine was found to have the highest binding affinity compared with ivermectin and remdesivir (Table 2). Intriguingly, ivermectin was found to be the best compound in binding the viral replicase (Table 2). Ivermectin-hACE2 was inferred as the weakest binding compared with that of hydroxychloroquine and remdesivir. On the other hand, interaction of ivermectin with human TMPRSS2 was found to be higher than remdesivir but lower to that of hydroxychloroquine (Table 2). Comparative simulation dynamics studies further supported the inferences drawn from molecular docking study and illuminated ivermectin as a potential anticorona compound (Figure 4).

**Table 2.** Comparative binding efficiency of ivermectin, remdesivir and hydroxychloroquine against the key proteins involved in SARS-CoV-2 pathogenesis.

Target protein	E_Tot_- value			
	Ivermectin		Remdesivir	^†^HCQ
	B1a	B1b		
**SARS-CoV-2**				
SARS-CoV-2 spike protein	-261.74	-287.0	-245.5	-812.00
SARS-CoV-2 Spike S1 receptor-binding domain	-372.99	-395.9	-353.1	+378.8
SARS-CoV-2 Spike S2 subunit	-393.29	-411.6	-340.8	-1301.2
SARS-CoV-2 main protease	-384.56	-408.6	-320.2	-981.8
SARS-CoV-2 NSP9 replicase	-327.47	-352.2	-312.55	+145.9
SARS-CoV-2 RNA-dependent RNA polymerase	-365.52	-428.6	-329.9	-1087.4
**Human**				
ACE2 receptor protein	-81.85	-91.4	-79.9	-1360.8
TMPRSS2	-392.75	-382.9	-313.30	-564.6

† Hydroxychloroquine.

### Binding energy of ivermectin B1b

We have determined the binding free energy of ivermectin B1b against RDRP as RDRP appears to be the most efficacious target of ivermectin. Free energy of binding at same pocket of RDRP with Ivm B1b is -9.67 kcal/mol and calculated binding affinity Kd is 76.89 nM (nanomolar).

### Comparative analysis of pharmacokinetic attributes of ivermectin, hydroxychloroquine & remdesivir

Analysis of ADME-Tox profile revealed that all the drugs have negative response to cytochrome P450 and p-glycoprotein inhibition and positive to human intestinal absorption. Lipophilicity value of ivermectin was found to be 5.74, which seems to be ideal for absorption and permeation. Skin permeability (logK_p_ is -7.14 cm/s) was found to be the highest while another important pharmacological property – drug likeness, was at par with remdesivir and better than hydroxychloroquine (Supplementary Table 2).

## Discussion

Ivermectin is a popular choice of drug for treating various parasitic infections till today. Since 1987, this drug has been used to treat more than 3.7 billion onchocerciasis patients through the Mectizan Donation Programme sponsored by Merck for eliminating of onchocerciasis [20]. Furthermore, lymphatic filariasis was included in this program in 1998 [21]. Ivermectin is also a member of the three-drug combinatorial therapy alongside the albendazole and diethylcarbamazine [22]. Ivermectin is also efficacious against the *Strongyloides*, scabies and soil-transmitted helminths [23]. Moreover, ivermectin has also been explored as an endectocide for reducing malarial vectors to reduce the disease transmission [24]. Ivermectin exerts its parasiticidal action through binding and blocking the anion/Cl^-^ ion channels located on the cell membrane, thereby causing disruption of the neuromuscular system leading to paralysis and death [23]. A major advantage of using this FDA-approved drug is its relatively benign nature at treatment doses in humans [25]. Recently, ivermectin has been reported for antiviral activity toward SARS-CoV-2 *in vitro* [13]. The study depicts that a low dose of ivermectin (5 micromolar) can induce 93% reduction in viral RNA from released virion and 99.8% reduction in cell-associated/unreleased virion after 24 h of incubation [13]. Interestingly, reduction of viral RNA was found to be increased up to 5000-times after 48 h of treatment [13]. Researchers have hypothesized that ivermectin binds and impairs Impα/β1 heterodimer, which plays a key role in binding the cargo protein of coronavirus and facilitates its translocation toward the nucleus [13]. Moreover, researchers have also claimed that ivermectin molecules may act as ionophores and be capable of producing osmotic lysis of the viral membrane [26]. Considering the high and rapid viricidal activity of ivermectin, involvement of a specific target is a question. Therefore, the present study was conducted *in silico* to explore the possible molecular targets of ivermectin in SARS-CoV-2 and the possible mechanism of interactions between ivermectin and the proteins involved in the viral pathogenesis. Such molecular interactions between ivermectin and the target proteins are most likely mediating the rapid and intense antiviral efficacy of ivermectin.

Spike glycoprotein has been the major viral molecule involved in binding host cell surface receptor and establishing infection [1]. Our molecular docking data and counter verification by molecular dynamic simulation collectively evidenced that ivermectin targets S2 subunit of spike protein and may cause conformational change, which may interfere with spike protein-ACE2 interaction (Figure 1A, Table 1 & Supplementary Table 1). SARS-CoV-2 uses a protease enzyme, namely chymotrypsin-like protease (3CLpro) or main protease (Mpro), which perform an important function to prime spike protein-mediated binding to human ACE2 and entry of the virus [3]. Herein, we checked the interaction between ivermectin and the viral protease and found a strong hydrophobic interaction between these two (Figure 1B & Table 1). Interestingly, the binding efficacy of ivermectin to SARS-CoV-2 replicase/RDRP was to found to be relatively high (Figure 2A–B & Table 1). In fact, ivermectin was found to be the best out of the three drugs in binding with viral replicase (Supplementary Table 2). Taking clue from the hypothesis on the effect of ivermectin on the viral targets, we further checked whether ivermectin could have any interaction with the relating partners present in the human. Earlier studies on the viral pathogenesis demonstrated the importance of human ACE2 receptor and TMPRSS2 proteins and for this reason the effect of ivermectin on these two targets were studied. We have documented a relatively weak binding of ivermectin B1A isomer with ACE2 from the biocomputational study (Supplementary Figure 3), orchestrated mainly by the residues of Asn61 and Asn64 on ACE2 (Supplementary Table 1). However, ivermectin B1B isomer was found to exert strong hydrogen bonding and hydrophobic interaction with ACE2 receptor of human (Supplementary Table 1). These data are encouraging as ACE2 mediates several important physiological functions in the human body including regulation of blood pressure and therefore an effect on this receptor could induce severe physiological imbalance. However, ivermectin was found to bind with TMPRSS2 with a better affinity and stability (Figure 2C).

In recent times, hydroxychloroquine has been tested for efficacy against SARS-CoV-2 [11] and it has been reported to inhibit the function of spike protein via binding with the sialic acid residue of membrane ganglioside [27]. On the other hand, remdesivir has also come out as another choice of drug for treating severe corona-patients and reported to inhibit replication of SARS-CoV-2 via direct binding with the viral RDRP [28]. But, this *in silico* study is a maiden report to compare the relative binding potential of these two drugs with ivermectin and presenting ivermectin as a potential anti-SARS-CoV-2 agent for its widespread use in the near future. In an earlier study, it has been clearly described that the inhibitory effect of ivermectin on SARS-CoV-2 is mediated by its direct binding to the active site residues of SARS-CoV-2 RdRp such as Ser759, Asp760 and -Asp761 that are present in the active site of the enzyme [29]. Interestingly, our data indicated that ivermectin strongly and stably binds with the viral replicase compared with hydroxychloroquine and remdesivir, while stability of ivermectin-viral protein complexes including S1 RBD, S2 protein, RDRP, TMPRSS2 was found to be better than that of remdesivir. The efficacy of ivermectin B1b has been found to be more than that of its B1a isomer. In recent times, studies on the comparative proteomics of ivermectin-treated SARS-CoV-2 and SARS-CoV-2-treated cell lines also supported the multitargeted action of ivermectin [30,31]. Eweas *et al.* [30] suggested a strong affinity of ivermectin toward *N* phosphoprotein and nsp14, which has been postulated to be involved in inhibiting the viral replication and assembly). A study conducted by Lehrer *et al.* [31] also provided evidence on the strong interaction of ivermectin with spike protein existing in a bound form with ACE2. A recent quantitative proteomics study by Li *et al.* [32] revealed alterations in the expression of 52 SARS-CoV-2/COVID-19-related proteins in ovarian cancer cell line after ivermectin treatment and such alterations in the proteins induced by ivermectin were found to influence the signaling event majorly involving cytokines and growth factor family, MAP kinase and G-protein family, and HLA class proteins. All these evidences support the findings of the present study indicating ivermectin as a broad-spectrum antiviral drug for treating COVID-19. Moreover, our *in silico* analyses on the pharmacokinetic profiles of these three drugs of interest also revealed ivermectin as a suitable drug candidate. As compared with hydroxychloroquine and remdesivir, ivermectin has relatively much higher water solubility and lipophilicity, further, having lesser skin permeation on the other hand (Supplementary Table 2). The three drugs included in the study are FDA-approved drugs and used for treating various parasitic (ivermectin and hydroxychloroquine) and viral (remdesivir) infections of human. However, to present the suitability of ivermectin for treating COVID-19, we have compared the pharmacological properties of ivermectin with the other two drugs.

Taken together, our data on the interaction between ivermectin and viral proteins indicated that ivermectin majorly acts by interfering with the viral entry through inhibiting the function of spike protein and protease. These studies also indicate that ivermectin may also target human ACE2 and TMPRSS2 for exerting its inhibitory action over SARS-CoV-2. However, all these *in silico* studies require subsequent experimental validation, which could enable Ivermectin as a drug of reliance to be used for counteracting the viral growth.

## Conclusion

Developing an effective therapeutic against COVID-19 is currently the utmost interest to the scientific communities. The present study depicts comparative binding efficacy of a promising FDA-approved drug, ivermectin, against major pathogenic proteins of SARS-CoV-2 and their human counterparts involved in host–pathogen interaction. Herein, our *in silico* data have indicated that ivermectin efficiently utilizes viral spike protein, main protease, replicase and human TMPRSS2 receptors as the most possible targets for executing its antiviral efficiency. Therefore, ivermectin exploits protein targets from both virus and human, which could be the reason behind its excellent *in vitro* efficacy against SARS-CoV-2 as reported by Caly *et al.* [13]. Ivermectin B1b isomers have been found to be the more efficacious molecule out of the two homologs. Intriguingly, comparison of the *in silico* efficiency of ivermectin with currently used anticorona drugs, such as hydroxychloroquine and remdesivir, indicated toward the potential of ivermectin to target the major pathogenic proteins of SARS-CoV-2. Ivermectin is a popular antiparasitic drug and is also safe in children, younger adults, pregnant and lactating ladies. Development of pulmonary delivery of ivermectin through synthesis of better ivermectin formulation has been reported recently and this is expected to shorten the treatment duration and lead to better outcomes [33]. It is noteworthy to mention that many anti-SARS-CoV-2s are now being tested for their efficacy in shaping the immune response of humans, through targeting the cell surface as well as intracellular toll-like receptors [34,35]. In this context, ivermectin could be an effective option as well. Considering all these facts, the present study explores the therapeutic targets of ivermectin against SARS-CoV-2 and enlightens the possibility of using this drug in COVID-19 clinical trials shortly.

**Summary points**

- The present *in silico* study presents the therapeutic efficacy of ivermectin against SARS-CoV-2 in comparison to two recently used anti SARS-CoV-2 drugs, namely remdesivir and hydroxychloroquine.
- Molecular docking was performed using the drugs of interest and various proteins involved in the infection cycle of SARS-CoV-2 such as spike glycoprotein, main protease, replicase, RNA-dependent RNA polymerase, human ACE2 receptor and human transmembrane serine protease. The dynamics of the interaction was further analyzed by molecular dynamics simulation studies and the binding free energy of binding of ivermectin to each protein was determined.
- The pharmacokinetic attributes of ivermectin were compared with other two anti-SARS-CoV-2 drugs and ivermectin was found to be a safe drug.
- Ivermectin was found to be an efficient inhibitor of Mpro, replicase and hTMPRSS2 and the study manifests a superior ground for the candidature of ivermectin to be an efficient anti-SARS-CoV-2 therapeutic option.
