# Supplementary material for: Exploring the binding efficacy of ivermectin against the key proteins of SARS-CoV-2 pathogenesis: an in silico approach
Source: Future Virol. 2021 Mar 25:10.2217/fvl-2020-0342. doi: 10.2217/fvl-2020-0342 (PMC7996102; doi:10.2217/fvl-2020-0342)

# Supplementary Documents

**Supplementary Tables**

**Table S1. Protein-ligand interactions for ivermectin-hACE2.**

| **Human ACE2 receptor protein** | | | | | | | |
| --- | --- | --- | --- | --- | --- | --- | --- |
| **Ivermectin B1A** | | | | **Ivermectin B1B** | | | |
| **Hydrophobic interaction** | | | | | | | |
| Residue | Distance (in Å) | Ligand Atom  No. | Protein Atom  No. | Residue | Distance (in Å) | Ligand Atom  No. | Protein Atom  No. |
|  | | | | ASN61 | 3.95 | 7449 | 416 |
| **Hydrogen bonding** | | | | | | | |
| Residue | Distance (in Å) | Ligand Atom  No. | Protein Atom  No. | Residue | Distance (in Å) | Ligand Atom  No. | Protein Atom  No. |
| ASN58 | 3.78 | 7448 | 388 | ASN61 | 4.05 | 7441 | 419 |
|  | | | | ASN64 | 2.31 | 7450 | 450 |

**Table S2. Comparative pharmacological profiles of ivermectin, hydroxychloroquine and remdesivir.**

| **Drugs** | **Water Solubility (Esol) log mol/lit** | **Lipophilicity (Log P_O/W_) iLOGP** | **Drug-likeness (Lipinski’s rule of five)** | **Skin permeation (logK_p_)** |
| --- | --- | --- | --- | --- |
| Ivermectin (IVM) B1A | -8.73 | 5.74 | 2 violation (MW-  875.09 g/mol, No. of H-bond acceptor- 14) | -7.14 cm/s |
| IVM B1B | -8.49 | 6.44 | 2 violation (MW-  861.07 g/mol, No. of H-bond acceptor- 14) | -7.30 cm/s |
| Hydroxychloroquine | -7.46 | 2.52 | 3 violation (MW-  864.76 g/mol, No. of H-bond acceptor- 18, No. of H-bond donor- 14) | -9.21 cm/s |
| Remdesivir | -3.49 | 3.73 | 2 violation (MW-  602.58 g/mol, No. of H-bond acceptor- 12) | -9.24 cm/s |

**Supplementary Figures**

Figure S1 **Structure of the drug and possible drug-targets against SARS-CoV-2.** A. Structures of Ivermectin isomers. B. Crystal structure of whole spike protein of SARS-Cov-2. C. Receptor binding domain (RBD) of S1 subunit and D. S2 subunit of spike protein. E. Native structures of SARS-CV-2 main protease. F. RNA-dependent RNA polymerase (Replicase) of SARS-CoV-2. Modelled structure of G. human ACE2 (hACE2) receptor protein and H. TMPRSS2.


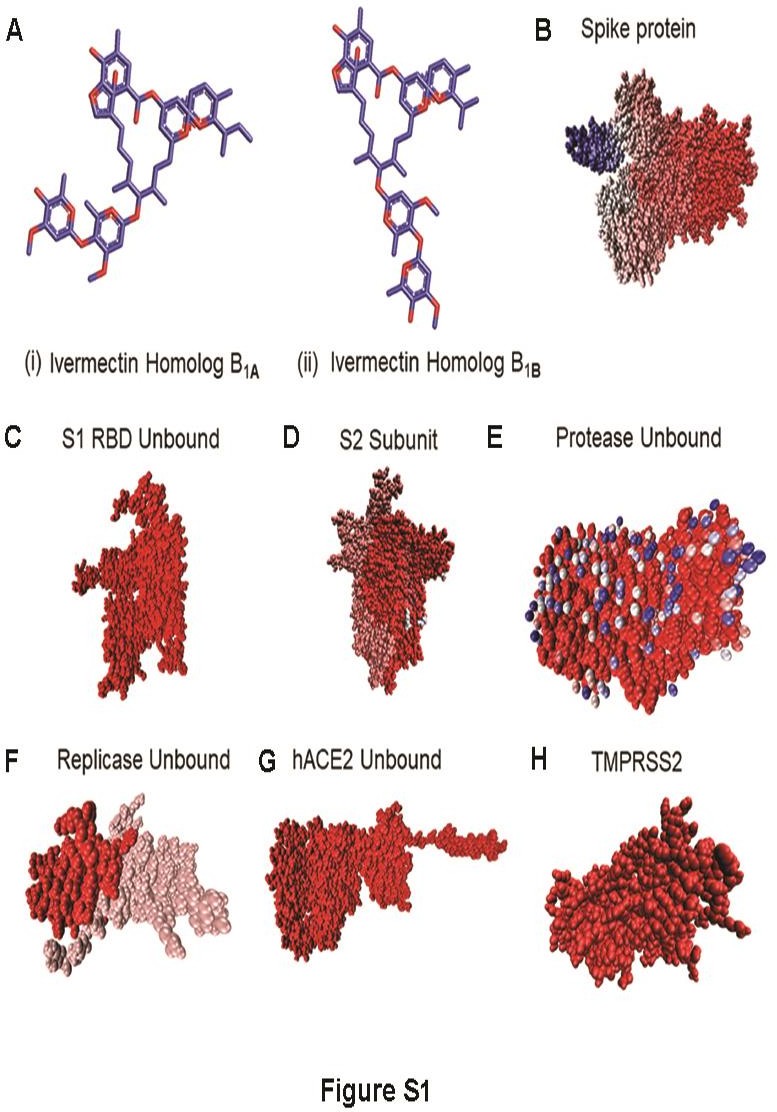


Figure S2 **Ramachandran plot showing stereochemical quality of** A. Receptor binding domain (RBD) of S1 subunit of spike protein and RNA-dependent RNA polymerase (Replicase) of SARS-CoV-2. B. S2 subunit of SARS-CoV-2 spike protein and C. Human TMPRSS2.


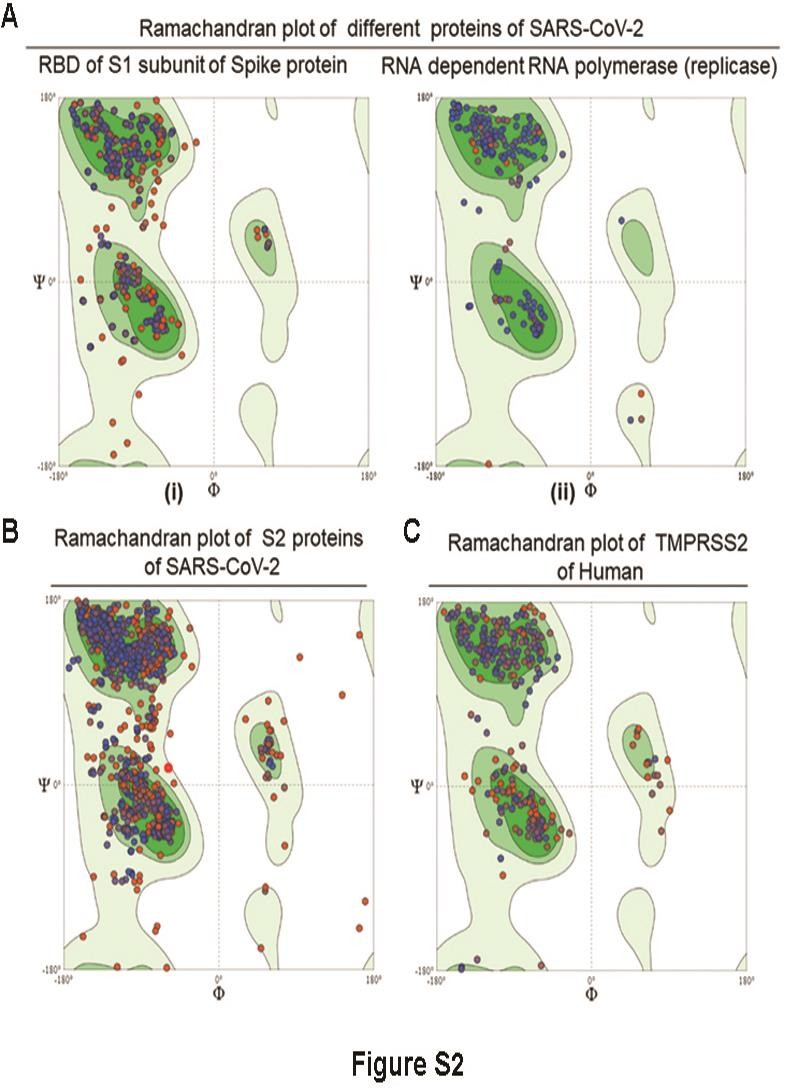


Figure S3 **Molecular interaction between ivermectin and human ACE2 (hACE2) receptor protein.** Binding of ivermectin (B1b) with hACE2 is showing A. Zoom out; B. Zoom in configuration and C. Cartoon model by space-filling models. D. hACE2-ivermectin interaction determined by PLIP. Solid red lines are showing the hydrogen bonds, while dotted red lines are showing the hydrophobic interaction.


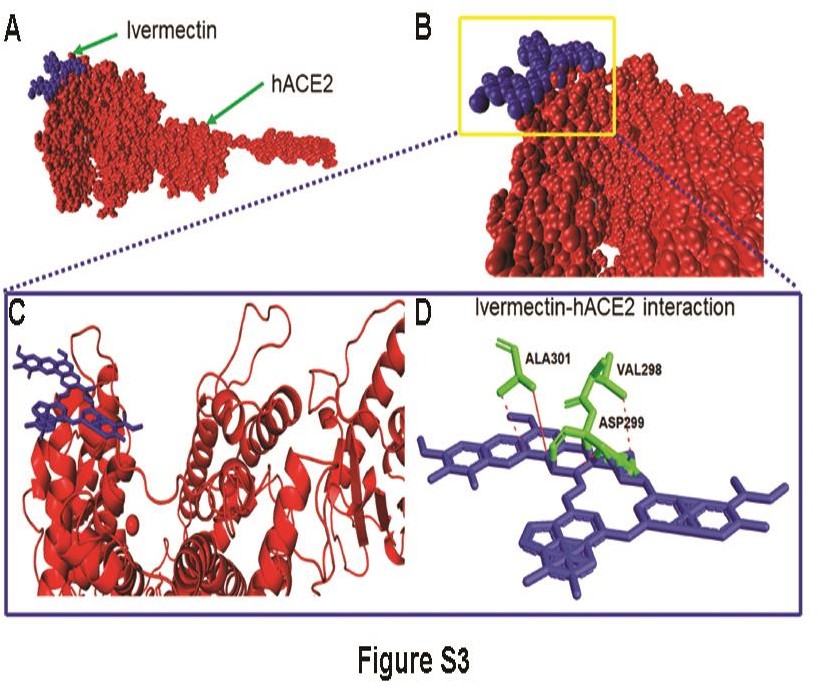


Figure S4

**Supplementary Fig. 4. Molecular dynamics simulation analyses of the ivermectin-replicase and ivermectin-RDRP interactions.** (A, B) Co-variance map (correlated (red), uncorrelated (white) or anti- correlated (blue) motions) and (C, D) elastic network (darker gray regions indicate more stiffer regions) of the complexes.


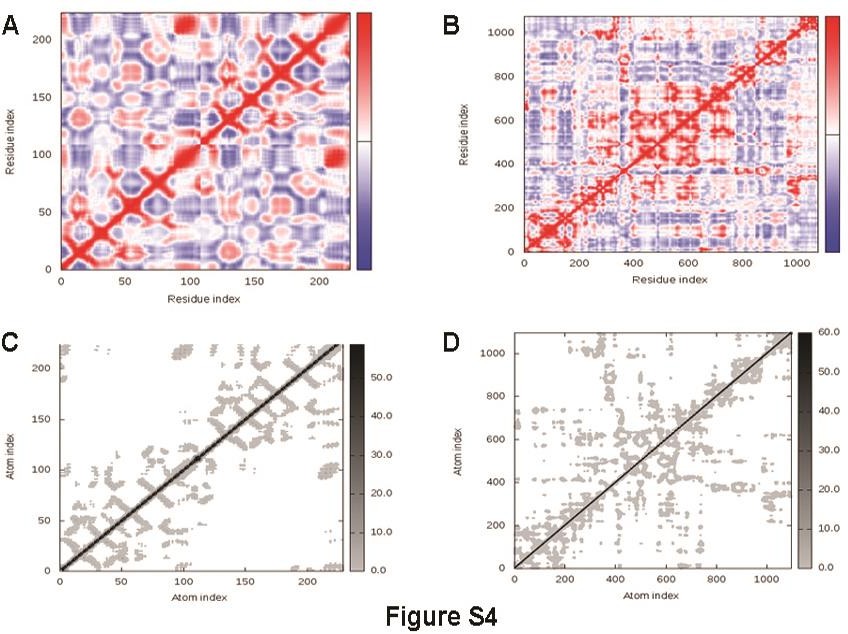

Supplement: Supplementary file 1 [file supplementary_materials.docx]
